# Sugarcoating it: Enterococcal polysaccharides as key modulators of host–pathogen interactions

**DOI:** 10.1371/journal.ppat.1009822

**Published:** 2021-09-09

**Authors:** Yusibeska Ramos, Stephanie Sansone, Diana K. Morales

**Affiliations:** 1 Department of Obstetrics and Gynecology, Weill Cornell Medicine, New York, New York, United States of America; 2 Department of Urology, Weill Cornell Medicine, New York, New York, United States of America; Nanyang Technological University, SINGAPORE

The human gastrointestinal tract (GIT) harbors a diverse microbial ecosystem that plays a critical role in defining health and disease. Members of the bacterial genus *Enterococcus* sp. are common gut-resident microbes that have the potential to cause life-threatening infections when GIT homeostasis is disrupted due to immunosuppression or prolonged antibiotic treatment [1]. *Enterococcus faecalis* and *Enterococcus faecium*, for instance, are predominant gut inhabitants accounting for the majority of hospital-acquired enterococcal infections [2–5]. The pathogenic success of enterococci is partly attributed to their intrinsic tolerance and acquired resistance to diverse antimicrobials, as well as their ability to endure and thrive in harsh environments [1]. Emerging studies suggest that this environmental persistence is in part mediated by polysaccharides [6], which generally consist of repeating units of diverse oligosaccharides covalently linked to the cell surface [7]. Of note, *Enterococcus* sp. possess highly diverse glycobiological arrangements (**Fig 1**) given their ability to build not only essential structural polysaccharides, such as peptidoglycan (PG), enterococcal polysaccharide antigen (EPA), and wall teichoic acids (WTAs), but also the capacity to synthesize capsule polysaccharides (CPs), lipoteichoic acids (LTAs), and other extracellular polysaccharides [7–11]. These glycans play a prominent role in maintaining bacterial cell integrity and morphology in conjunction with building a dynamic interface with the environment [11,12]. Enterococcal polysaccharides also coordinate major host–pathogen interactions since they modulate cell adhesion and the formation of microbial multicellular communities. Further, these complex sugars have been proposed to promote GIT colonization and penetration of intestinal barriers [13–16] while enhancing resistance to phages, antibiotics, and even the host’s immune system [17–24]. In this review, we discuss multiple mechanisms through which polysaccharides can shape the physiology and pathogenicity of enterococci, with an especial emphasis on *E*. *faecalis* and *E*. *faecium*.

**Fig 1:**
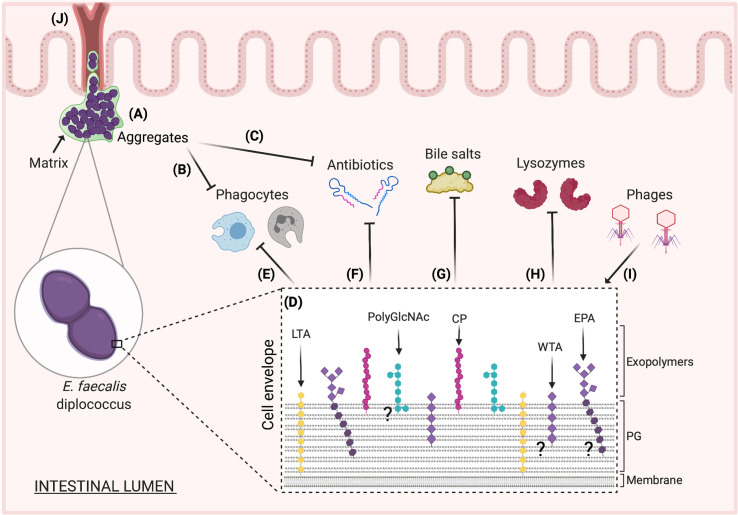
Proposed physiological roles of *Enterococcus faecalis* polysaccharides in the gut. **(A)***E*. *faecalis* intestinal colonization has been proposed to be mediated by the formation of multicellular aggregates covered with and connected by a matrix (green) partly composed of exopolysaccharides [34,69]. **(B)** Formation of these complex communities may allow enterococci to evade the immune system by promoting survival within phagocytes (macrophages or neutrophils) and **(C)** to resist the effect of antibiotics produced by other gut bacteria. **(D)** Cells within these complex communities possess a variety of glycopolymers that promote aggregate formation and structural development [25]. Among these polymers, PG creates a layer that protects cells from osmotic pressure, reinforces cell shape and size, and shelters cell envelope components, including other structural/nonstructural polysaccharides [28]. The proposed scheme was based on previous publications [7,10,11] and shows an enterococcal cell envelope with relative glycobiological arrangements and positions. These glycopolymers, from left to right, include the membrane-anchored LTA (yellow), the exopolysaccharides containing polyGlcNAc (cyan), the covalently PG-bound CP (fuchsia), the WTA (light purple) that may also be bound to PG, and the EPA, which possesses a rhamnan backbone (dark purple) possibly anchored to PG and extracellular exposed WTA decorations (light purple). **(E)** WTAs, LTAs, EPA, and/or CPs allow *E*. *faecalis* to evade phagocytosis by macrophages or neutrophils [24,76,80,81,84–86]. These cell envelope components likely act as a shield that prevent complement detection and mediate resistance to phagocytosis. Changes to these polysaccharides may disrupt the cell envelope topography, leaving the bacteria naked or likely exposing “neoepitopes” for recognition by the complement system. **(F)** Changes in PG and EPA structure or composition may also be contributing factors in the resistance to antibiotics, such as those targeting the enterococcal cell envelope [13,17,20,24,53,55,57]. **(G)** *E*. *faecalis* may resist the intestinal bile salt toxicity through rearrangements in the composition of EPA and/or PG that counteract the detergent activity of these amphipathic molecules and the induced external osmotic pressure. **(H)** This bacterium can evade the immune response and persist in the gut by resisting the antimicrobial effect of lysozymes through modifications of their PG structure [81,95–97]. **(I)** EPA also acts as a “receptor” that is recognized by phages during viral infection, and changes in EPA decoration can confer resistance to these phages by *E*. *faecalis* [18,47]. **(J)** Upon intestinal overgrowth, this bacterium can exit the intestinal lumen to reach the bloodstream and colonize distal anatomical sites. The formation of matrix-covered enterococcal aggregates may represent a new mechanism that promotes *E*. *faecalis* translocation across the gut epithelial barriers in susceptible hosts, where EPA or/and polyGlcNAc-containing exopolymers may facilitate this migration. (?) denotes that the exact localization of EPA, polyGlcNAc-containing polysaccharides, and WTA in the enterococcal cell envelope is uncertain. CP, capsule polysaccharide; EPA, enterococcal polysaccharide antigen; LTA, lipoteichoic acid; PG, peptidoglycan; polyGlcNAc, β-(1,6)-GlcNAc polymers; WTA, wall teichoic acid.

## Polysaccharides in the development of complex multicellular communities

Enterococci form structurally complex communities (biofilms) in a number of infections, where they become not only difficult to eradicate, but also a source of bacterial dissemination and a reservoir for antibiotic resistance [25]. Biofilms mainly consist of a population of cells bound together and embedded in a self-produced extracellular matrix [26]. The process of biofilm formation generally involves initial surface attachment, aggregate (microcolony) development, structural maturation (or biofilm growth), and cell dispersal [25]. While the role of polysaccharides in biofilm formation has been extensively delineated in other bacterial species [26,27], little is known about how these glycans affect the development of enterococcal communities. Below, we summarize recent findings highlighting the critical role of polysaccharides in enterococcal biofilms:

### Cell and surface attachment

Enterococcal glycopolymers, such as PG, LTAs, EPA, and other exopolysaccharides, contribute to the initiation of biofilm formation. PG, which consists of a polymeric mesh (PG sacculus) with strands of repeating β(1,4)-linked *N*-acetylglucosamine (GlcNAc) and *N*-acetylmuramic acid (MurNAc) disaccharides cross-linked through short peptide stems, can undergo constant remodeling in response to changing environmental conditions [28]. Hence, during biofilm growth, it has been shown that *E*. *faecalis* alters its PG through modifying its peptide stems, increasing cross-linking, *N*-deacetylation, and reducing O-acetylation, in comparison to PG extracted from free-living (planktonic) enterococcal cells in vitro [8]. These changes may impact PG’s interactions with other ions and the overall cell charge, which modifies *E*. *faecalis*’ ability to attach to surfaces and enhances cell-to-cell aggregation for biofilm formation. PG also contributes to maintaining cell shape and serves as a scaffold for other components involved in attachment and biofilm formation, including cell wall–anchored LPxTG surface proteins, such as enterococcal surface protein (Esp), and the endocarditis and biofilm-associated pili (Ebp) [29–31]. Along with surface proteins, WTAs, LTAs, CPs, and the EPA polymer are sheltered by the PG layer (**Fig 1**).

**Fig 2:**
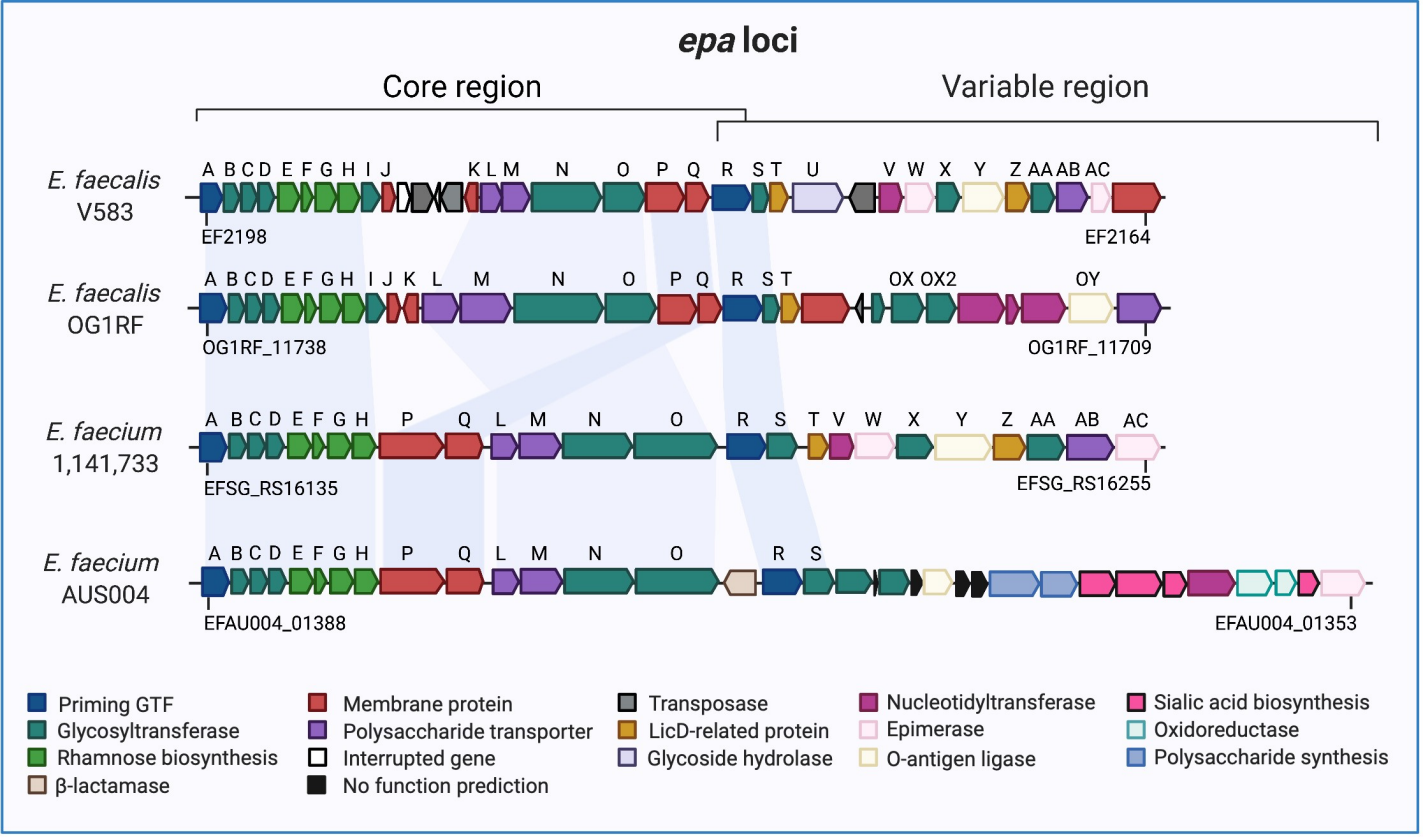
Comparison of predicted *epa*-like loci among enterococcal strains. In *Enterococcus faecalis* V583 and OG1RF, EPA biosynthesis is encoded by a cluster of genes organized in 2 genetic loci: first, a conserved region, consisting of 18 genes, from *epaA* to *epaR*, which participate in the rhamnan backbone biosynthesis (core region), and, second, a downstream cluster of approximately 10–20 genes exhibiting genetic variability among strains, which has been proposed to account for the major differences on EPA decoration among *E*. *faecalis* isolates (variable region) [10,14,32,105]. The core region of *Enterococcus faecium* is differentially organized in comparison with the EPA core from *E*. *faecalis*. It does not have homologs of *epaI*, *epaJ*, and *epaK*. Instead, it has the 2 genes, *epaP* and *epaQ*, located at that site [104,105,107]. *E*. *faecium*’s variable locus is proposed to be divided into 4 main variants based on sequence similarities [104,107]. The scheme depicts variants 2 and 4 for strains 1,141,733 and Aus0004, respectively. Arrow colors correspond to colored boxes (bottom) and indicate predicted open reading frame function according to genome annotations and BLASTP analysis. Blue shades connect homologs of different strains. The absence/variability of *epa* genes suggests that the EPA polysaccharides of the 2 species have different sugar compositions, and, in consequence, may confer diverse physiological functions. The *epa* loci were adapted from [10,20,32,54,104,105,107]. All genes are drawn to scale. EPA, enterococcal polysaccharide antigen; GTF, glycosyltransferase.

LTAs have also been shown to promote the initial steps of biofilm formation, with the interaction of these long anionic polymers with cell surface adhesins facilitating the attachment of *E*. *faecalis* to itself and/or other surfaces such as epithelial tissues [9]. Furthermore, different modifications in the carbohydrate composition of EPA, a rhamnan polymer backbone (EPA core) that binds to cell wall–exposed WTAs (ribitol teichoic acids; EPA decorations) [10], alter surface hydrophobicity and charge, thus affecting cell-to-cell attachment [22,24]. Several biofilm-related phenotypes have been attributed to this polymer. For instance, while Teng and colleagues reported that mutations in genes encoding the EPA core (*epaA*, *epaB*, *epaE*, *epaN*, and *epaM*; **Fig 2**) reduced *E*. *faecalis* OG1RF biofilm formation [32,33], recent studies using the same parental strain demonstrated that mutants involved in either EPA backbone synthesis (Δ*epaI* and Δ*epaQ*) or EPA decoration (Δ*epaOX*) decreased biofilm biomass only when grown in different settings with subinhibitory concentrations of antibiotics [20,22]. Moreover, strains carrying insertion mutations in *epaJ* or *epaK* showed no phenotypic differences compared with wild-type (WT) biofilms in the described assays [22]. Consistent with these results, it has been demonstrated that biofilm formation is influenced by the growth medium and nutrient availability [34–36] and that the media composition can also affect the polysaccharide profile of a mutant altered in decorating its EPA backbone [14,15]. Hence, these observations suggest that EPA may be modified in response to external growth conditions, impacting enterococcal biofilm development in multiple ways.

### Microcolony formation and biofilm maturation

After initial attachment, the continuous growth of a surrounding matrix composed of exopolysaccharides and other macromolecules promotes cell aggregate/microcolony formation and biofilm growth [37]. The EPA polysaccharide, for instance, has been shown to define the *E*. *faecalis* biofilm architectural arrangement by stabilizing the maturation of the transient aggregates [22,38]. Indeed, deletion of genes in the core or variable region of the *epa* loci (**Fig 2**) affects *E*. *faecalis* cell shape, polysaccharide composition, and membrane potential [14,15,22,38]. This, in turn, promotes the formation of microcolonies that exhibit reduced structural integrity, compared with parental biofilms [22,38], likely due to induced changes in enterococcal adhesion traits. *E*. *faecalis* biofilm architecture has been found to be altered by specific *epa* mutations [20,22]. In contrast to WT (strain OG1RF) or Δ*epaOX*, which formed biofilms consisting of monolayers or clumped cells and long chain areas, respectively [20,22], Δ*epaQ* developed thicker structures of combined architectures including cells arranging in monolayers and large aggregates [22]. While the chemical nature of the exopolysaccharides composing the enterococcal matrix is still unclear, our group identified by immunostaining that *E*. *faecalis*’ aggregates are covered by exopolysaccharides formed partly by polymers of β-(1,6)-GlcNAc (polyGlcNAc) [15]. Although the precise structure of these polysaccharides has not been fully elucidated, we demonstrated that the deletion of *epaX* in *E*. *faecalis* V583, a gene involved in EPA decoration (**Fig 2**) [10], caused a marked reduction in the matrix surrounding the aggregates [15]. Notably, this defect correlated with the lack of detection of polyGlcNAc-containing polymers by antibody-based methods, in comparison with the parental counterpart [15]. Studies by Guerardel and colleagues did not find detectable amounts of polyGlcNAc-containing polymers in planktonic cultures of *E*. *faecalis* [10], indicating that the culture conditions may account for such observations by impacting *E*. *faecalis* exopolysaccharide production. In fact, it has been reported that the exopolymers’ chemical composition can be altered in response to the specific nutrients supplemented [39]. Future work using different growth conditions may help explain the link between *epa*X and the production of the polyGlcNAc-containing exopolysaccharides, which might be involved in polyGlcNAc synthesis and/or contribute to retention or binding of the exopolymer through negatively charged EPA decorations. Since the absence of polyGlcNAc-like exopolysaccharides decreases the ability to develop structured biofilms in other gram-positive bacteria [40,41], additional biophysical and chemical analyses are warranted to comprehensibly understand the role of enterococcal polyGlcNAc-containing exopolymers in aggregate/biofilm formation and its matrix physiology.

## Polysaccharides in enterococcal adaptation to harsh conditions

Bacteria have evolved sophisticated mechanisms to survive in hostile environments, including the human body. Polysaccharides provide an adaptive advantage to cope with such external stressors. In biofilms, for instance, modifications of exopolysaccharides during glucose starvation increased *E*. *faecalis*’ hydrophobicity, enabling survival in energy-depleted environments such as root canals [42]. Moreover, *E*. *faecium* probiotic strains have been shown to produce exopolysaccharides that can scavenge reactive oxygen species in vitro, suggesting a potent antioxidant defense mechanism mediated by these glycopolymers [43]. Below, we summarize how polysaccharides promote enterococcal resistance to some environmental stressors.

### Resistance to osmotic stress

EPA serves a protective role for enterococci in the GIT where bile salts disrupt bacterial membranes and cause osmotic stress [44]. Intestinal bacteria resist bile by activating efflux systems and remodeling their cell envelope [45]. Under high osmolarity, *E*. *faecalis* is capable of up-regulating expression of *epa* genes, especially those that code for rhamnose biosynthesis and glycosyltransferases. For example, mutants in the *epa* core region (**Fig 2**), Δ*epaB* and Δ*epaR*, exhibit increased susceptibility to salt-induced osmotic stress [46,47]. Moreover, the absence of EpaOX considerably affects cell envelope integrity [38] by compromising decorations of rhamnan backbone required for optimal polymerization of PG by penicillin-binding proteins [24]. When intact, the PG sacculus protects cells from osmotic rupture and reinforces cell shape and size [28]. Indeed, strains Δ*epaOX* in OG1RF and Δ*epaX* in V583 showed an altered cell morphology and an increased sensitivity to bile salt components [14,20], suggesting that EPA and PG may play an important role during the remodeling of the cell envelope to counteract changes in the external osmolarity in enterococci.

### Resistance to antibiotics

Polysaccharides are also key players in mediating resistance to antibiotics. PG is a major target for many antimicrobials in bacteria including β-lactams and glycopeptides (vancomycin). Enterococci have evolved multiple mechanisms to counteract these antibiotics (for comprehensive reviews, see [48,49]). *E*. *faecium*, for instance, modifies its PG intermediaries by replacing the D-alanine–D-alanine (vancomycin’s target) with D-alanine–D-lactate as the terminal amino acids in lipid II [50]. This modification decreases the interaction between vancomycin and PG due to loss of a single hydrogen bond, conferring high levels of resistance to this bacterium [50]. *E*. *faecalis*, on the other hand, synthesizes vancomycin-resistant PG precursors terminating in D-alanine–D-serine [51]. The additional hydroxyl group of D-serine also introduces a steric hindrance for interaction with vancomycin, lowering this antibiotic affinity for PG [52,53]. Many of these glycopeptide resistance mechanisms in these strains are encoded in transposons or plasmids and involve the concerted action of multiple enzymes altering the basic structure of the cell envelope to overcome the damaging action of vancomycin [48,49].

Changes in the composition of EPA caused by mutations in the *epa* loci have also been shown to decrease sensitivity to antibiotics that target cell envelope synthesis, such as ceftriaxone or carbapenems in agar plates with Etest strips [54,55]. This adaptation is thought to result in part from reduced rhamnose and/or increased mannose abundance in some of these strains’ cell wall polysaccharides [55]. Moreover, a prior study reported that changes in the minimal inhibitory concentration between parental and *epa* core mutants were modest in agar plates with daptomycin using Etest strips [55], whereas Dale and colleagues showed that the deletion of *epaI* (EPA core) and *epaOX* resulted in enhanced susceptibility of *E*. *faecalis* OG1RF planktonic cells to the same antibiotic [20]. These contrasting findings suggest that the growth conditions used in both studies may differentially affect EPA synthesis/composition, and, thus, the cell envelope integrity necessary to prevent daptomycin from reaching the division septum. Reinforcing the role of EPA in modulating enterococcal resistance to antimicrobials, a recent study demonstrated that mutations in *galU*, which encodes UTP-glucose-1-phosphate uridylyltransferase (an enzyme involved in generating the UPD glucose required for *E*. *faecalis* galactose fermentation), affects EPA synthesis and changes the enterococcal susceptibility to β-lactams in agar dilution assays [56]. Additional studies have demonstrated that the EPA confers protection to multiple antibiotics, including gentamicin, vancomycin, and fusidic acid exposure under distinct growth conditions [13,17,20,55,57]. While informative, further research considering environmental factors and/or culture settings that may impact polysaccharide composition is needed to understand the precise mechanisms that mediate EPA-driven antibiotic resistance in enterococci.

Enterococci can also acquire antibiotic resistance by forming biofilms. Several characteristics of these structures contribute to enhance antibiotic resistance relative to planktonic cultures, including poor antibiotic penetration into the biofilm, antibiotic sequestration, and the presence of persister cells within the biofilm (for an excellent review, see [25]). Although *E*. *faecium* is intrinsically more resistant to antibiotics, *E*. *faecalis* forms thicker biofilms that promote tolerance to antimicrobials [58,59]. It has been previously reported that certain genes of the *epa* loci contribute to biofilm-associated antibiotic resistance [20,22,38]. Mutations in *epaI*, *epaQ*, and *epaOX* have demonstrated reduced biofilm formation in the presence of daptomycin, and only the latter showed to be more sensitive to gentamicin exposure. In sharp contrast, all 3 deletion mutants showed increased resistance to ceftriaxone [22]. Resistance to this antibiotic involves 2 interacting signaling pathways: the eukaryotic-like Ser/Thr kinase ireK and the CroS/R 2-component regulatory system [60–62]. IreK is considered to monitor the cell wall integrity and respond to cell wall stress [63]. Hence, EPA alterations resulting in cell wall and shape changes [14,20,22], especially in biofilms, may lead to IreK activation of this pathway, leading to a higher intrinsic resistance to ceftriaxone. In fact, dysregulated IreK or CroS/R pathways (like strains lacking negative regulators for these systems) display increased cephalosporin resistance [64,65]. Further analysis to establish the connection between the cephalosporin resistance phenotype of *epa* mutants and the IreK and CroS/R pathways could provide insights into the cell envelope stress signals detected by these systems.

### Resistance to bacteriophages

To infect bacteria, phages must bind to cell wall–associated molecules, such as polysaccharides, which serve as receptors during viral infection [66]. EPA can operate as one of these “receptors,” coordinating phage absorption and subsequent infection by lytic *E*. *faecalis* phages [17]. The ability for phages to infect *E*. *faecalis* depends specifically on certain genes of both the core and the variable region of the *epa* loci [18,47]. Mutations in these genes have been found to promote phage resistance, but they also increase *E*. *faecalis’* sensitivity to osmotic stress and antibiotics that target the cell wall [17,18,47], suggesting that phage resistance in enterococci might be a fitness trade-off. Furthermore, in *E*. *faecium*, it has been reported that changes in the composition of cell wall polysaccharides like CPs and EPA promote phage resistance adaptation. Notably, mutations in *epaR* and *epaX* limit phage adsorption to *E*. *faecium*, although to a lesser extent than similar mutations in *E*. *faecalis* [54]. These adaptive traits act as gatekeepers to phage absorption, conferring additional means through which *Enterococcus* thrives.

## Glycopolymers in GIT colonization, exit, and distal spread

Enterococci have been found in the intestinal lumen as well as in the complex mucus layer and crypts of the small intestine [67,68]. GIT colonization by *E*. *faecalis* seems to be mediated by the formation of microcolonies rather than structured biofilms, as evidenced by the formation of enterococcal aggregates covered by a fibrous, sweater-like, matrix predominantly localized throughout the GIT of germ-free mice [34]. Similarly, McKenney and colleagues demonstrated that vancomycin-resistant *E*. *faecium* substantially increased the formation of aggregates in the cecum of antibiotic-treated mice supplemented with lithocholic acid. This secondary bile acid, prevalent in the intestine of mice and humans, impaired the separation of growing enterococcal diplococci, causing the formation of long chains in vitro. Thus, mutants locked in the diplococci state were deficient in mouse tissue colonization and persistence, likely due to their inability to form aggregates and compete with the intestinal microbiota [69].

In addition, it has been demonstrated that EPA plays a role in GIT colonization. Studies involving the deletion of *epaX* and *epaS*, or the entire loci variable region, showed decreased intestinal colonization by *E*. *faecalis*, as evidenced by lower colony-forming units obtained from stool samples of mice treated with the mutants relative to WT V583 [13,14,17]. Notably, Δ*epa*S could not overgrow upon antibiotic-driven dysbiosis and failed to be efficiently transmitted to young mice following birth [17], suggesting that EPA promotes *E*. *faecalis* aggregate stability during GIT colonization. Indeed, loss of a glycosyltransferase from the *epa* variable loci reduces biofilm formation in the presence of sodium cholate (a bile acid component), and aggregates formed by a mutant in this locus exhibit reduced structural cohesion in vitro [20,38]. Although it has been observed that the aggregates formed by *E*. *faecalis* in vivo are morphologically similar to those formed in continuous flow bioreactors [34,38], the determinants of microcolony structural development in vivo are poorly characterized. Therefore, analyses to determine the role of EPA in aggregate formation/architecture using microscopic imaging of the GIT in murine models deserve further attention.

Upon damage of the gut barrier, enterococcal overgrowth in the lumen can promote breaching of the intestinal epithelium, a process known as translocation [70–72]. Some intrinsic factors have been proposed to contribute directly or indirectly to enterococcal egress from the GIT [15,16,73,74]. Among them, genes in the core and variable region (*epaX*) of the *epa* loci have shown to be necessary for efficient migration through intestinal epithelial barriers in vitro [15,16]. Moreover, using immunofluorescence microscopy, we found that *E*. *faecalis* WT formed aggregates covered by polyGlcNAc-containing exopolysaccharides that localized with the epithelial actin cytoskeleton during translocation. These polymers, however, were not detected when Δ*epaX* strains were used in the same experimental setting [15]. Hence, formation of matrix-covered enterococcal aggregates might represent a new mechanism that promotes colonization and/or migration across the intestinal barriers in susceptible hosts. Further studies using in vivo models are needed to gain a deeper knowledge of the role of EPA and other uncharacterized enterococcal polysaccharides in bacterial translocation through the intestinal epithelium, a key step for switching from gut commensal to pathogen.

## Enterococcal polymers facilitate immune resistance and evasion

Once outside the intestine, enterococci can spread and colonize other body sites, where they can become drivers of disease. Both as successful inhabitants of the GIT and potential pathogens, enterococci have developed mechanisms partly mediated by their cell surface polysaccharides to modulate, resist, or evade the host innate immune system (see review for further mechanisms [23]). The host immune response involves physical and chemical barriers, including soluble proteins, pattern recognition receptors, and phagocytes. The formation of biofilms has been proposed as a key strategy used by enterococci to evade the immune system. For instance, when the host epithelial barrier is disrupted, *E*. *faecalis* has been found to form matrix-encased microcolonies that can be internalized and encapsulated by the wounded tissue. In these settings, reduced cytokine and chemokine induction has been evidenced, suggesting that this bacterium can modulate the immune response to promote its persistence in wounded tissues [75]. Consistent with this finding, previous studies demonstrated that *E*. *faecalis* biofilms induce lower pro-inflammatory responses and promote survival within phagocytes in comparison with planktonic cells in vitro [76–79]. Thus, biofilms may be better adapted to overcome host defenses in vivo. Further analyses to determine the role of enterococcal polysaccharides in biofilm-mediated immune resistance using animal models deserve attention.

Several intrinsic components of the enterococcal cell envelope also play important roles during these host–pathogen interactions (**Fig 1**) [24,80]. For example, an *E*. *faecalis* deletion mutant defective in LTA D-alanylation demonstrated lower capacity to form biofilms and greater antibody recognition, which is an integral part of the humoral immune response capable of blocking adhesion and entry of a pathogen into tissues while promoting opsonophagocytosis [76,81]. Enterococcal LTA has also been proposed to alter macrophage function by promoting autophagy, a lysosome-mediated degradation process of internal cell components [82]. The LTA of *E*. *faecalis* can also inhibit Toll-like receptor (TLR)-mediated responses in periodontal tissues, which may represent an additional mechanism of macrophage suppression [83]. Disruption of *epa* core genes result in “naked” enterococcal cells lacking the EPA polymer that are more sensitive to killing by phagocytes [84]. While the mechanisms behind this process remain elusive, it is likely that changes in the carbohydrate composition of these mutants may expose “neoepitopes” for recognition by the complement system. However, it is unclear whether EPA is directly recognized by immune cells or it encases other cell factors to prevent *E*. *faecalis* phagocytic clearance. Additional research then demonstrated that EPA decorations also aid in protection against complement-mediated phagocytic killing [24], suggesting that modifications in EPA, like the covalent link of 2 ribitol-containing WTAs [10], rather than the backbone itself, support phagocyte evasion. Consistent with these findings, mutations in *tagB* resulting in the loss of these 2 ribitol-containing WTAs and secondary changes in EPA also led to increased complement recognition and augmented phagocytic killing of *E*. *faecalis* in vitro [85]. It is conceivable that the absence of WTAs in these mutants could disrupt the cell envelope topography, rearranging the surface localization of EPA, LTAs, and/or CPs. Hence, WTAs may mediate resistance to phagocytosis indirectly by acting as a shield for other surface glycopolymers and preventing complement detection [80,81,85,86]. Whether *E*. *faecalis*’ ribitol teichoic acids are only linked to EPA backbone or also covalently attached to MurNAc on PG as described in other gram-positive bacteria [87,88] deserves further investigation.

Enterococci are also capable of evading the immune system by resisting the antimicrobial effect of lysozymes, which are critical components of the host innate immune response. In mammals, these enzymes are found abundantly in the blood, liver, secretions, phagocytes, and at mucosal surfaces [89–92]. Lysozymes can hydrolyze the β-(1,4) glycosidic bonds linking PG monosaccharides (muramidase activity) and/or bind and create pores in negatively charged bacterial membranes, acting as cationic antimicrobial peptides (CAMPs) [93,94]. These lysozyme-dependent effects kill bacteria and release immunomodulatory bacterial components, including PG fragments [89]. *E*. *faecalis* has developed mechanisms to counteract lysozymes, including modifying its PG structure through GlcNAc deacetylation, MurNAc O-acetylation, or/and reduction of the cell net negative charge with the addition of positively charged D-alanine residues to WTA and LTA [81,95–97]. Recent studies have specifically shown that exposing *E*. *faecalis* to lysozyme triggers the production of a PG deacetylase that contributes to this bacterium’s virulence in the *Galleria mellonella* insect model [97]. Enterococci are also exposed to other CAMPs, which are positively charged products secreted by host cells or intestinal microflora. Mutations that increase the cell envelope’s negative charge (deletion of *tagB*, required for WTA biosynthesis) affect LTA D-alanylation (deletion of *dltA*) or/and alter EPA decoration (*epaX*-like mutations) result in increased sensitivity to CAMPs by *E*. *faecalis* [24,81,85].

CP expression is another strategy that allows enterococci to evade the immune system. These glycopolymers are localized at the cell surface and function as shields to mask underlying cell surface structures to reduce opsonization [98]. In *E*. *faecalis* and *E*. *faecium*, insertional inactivation of genes in the CP biosynthetic pathway yielded mutants with enhanced susceptibility to phagocytic killing by neutrophils and compromised *Enterococcus’* ability to persist in regional lymph nodes [99,100]. *E*. *faecalis* specifically produces 2 CP serotypes (C and D) with higher resistance to complement-mediated opsonophagocytosis than unencapsulated strains [101], contributing to innate immune evasion and increased pathogenesis [102]. These differences observed in opsonophagocytosis have been attributed to the ability of CPs to mask the detection of LTAs and surface bound C3 (a complement component required for activation of this pathway), thus preventing macrophage activation and reducing pro-inflammatory cytokine production such as tumor necrosis factor α [101,103]. However, to date, the chemical structures of these CPs have not been published. On the other hand, genome sequencing analyses revealed that, although several *E*. *faecium* strains do not possess *cpsC*-*cpsK* homologs required for CP synthesis in *E*. *faecalis*, a putative CP biosynthetic region is conserved among all enterococcal species except *E*. *faecalis* [101,104,105]. Notably, in vancomycin-resistant *E*. *faecium*, mutations in this novel locus correlate with enhanced biofilm formation, antibiotic tolerance, and lysozyme resistance in vitro [106], suggesting that the absence/modification of CP may confer a fitness advantage to *E*. *faecium* in the host.

Enterococci have therefore adopted multiple strategies to evade immune surveillance, contributing to its successful transition to a resilient pathogen. It is evident that polysaccharides influence their physiology by enhancing enterococci’s ability to form multicellular aggregates, colonize the GIT and extraintestinal sites, endure environmental stressors, evade the host immune system, and resist antibiotics. Upon loss of functional WTAs, LTAs, CPs, or EPA genes, *Enterococcus* sp. lose these remarkable capabilities. Clearly, the diversity of polysaccharides able to be produced in different growth conditions or by strains/species is likely to influence enterococcal fitness and survival. Examples of this diversity are reflected in the differences found in the variable region of the *epa* loci that may change the EPA decoration or structure (**Fig 2**). Thus, enterococcal polymers represent a high-profile target for future therapeutics, potentially leading to new strategies to effectively control systemic and persistent enterococcal infections. Understanding the vastly unexplored connections between the host, gut microbiota, and enterococcal polysaccharides will provide “sweet” insights into the mechanisms of enterococcal colonization and pathogenesis.
